# Small-scale pig farmers’ behavior, silent release of African swine fever virus and consequences for disease spread

**DOI:** 10.1038/srep17074

**Published:** 2015-11-27

**Authors:** Solenne Costard, Francisco J. Zagmutt, Thibaud Porphyre, Dirk Udo Pfeiffer

**Affiliations:** 1 Veterinary Epidemiology, Economics and Public Health Group, Royal Veterinary College, Hawkshead Lane, North Mymms, AL9 7TA, UK; 2 EpiX Analytics, 1643 Spruce St, Boulder, CO 80302, USA; 3 Centre for Immunity, Infection and Evolution, University of Edinburgh, Kings Buildings, Charlotte Auerbach Road, Edinburgh, EH9 3FL, Scotland, UK

## Abstract

The expanding distribution of African swine fever (ASF) is threatening the pig industry worldwide. Most outbreaks occur in backyard and small-scale herds, where poor farmers often attempt to limit the disease’s economic consequences by the emergency sale of their pigs. The risk of African swine fever virus (ASFV) release via this emergency sale was investigated. Simulation modeling was used to study ASFV transmission in backyard and small-scale farms as well as the emergency sale of pigs, and the potential impact of improving farmers and traders’ clinical diagnosis ability–its timeliness and/or accuracy–was assessed. The risk of ASFV release was shown to be high, and improving farmers’ clinical diagnosis ability does not appear sufficient to effectively reduce this risk. Estimates obtained also showed that the distribution of herd size within the backyard and small-scale sectors influences the relative contribution of these farms to the risk of release of infected pigs. These findings can inform surveillance and control programs.

African swine fever (ASF) is a viral disease with severe consequences, and its expanding distribution threatens the pig industry in the European Union and worldwide^1^,^2^,^3^,^4^. Once introduced to a new territory, the disease is difficult to control due to its high potential for spread, and the absence of vaccines or treatment. Strategic prevention, emergency preparedness and response programs are thus very important for ASF-free countries.

The disease is endemic in most of Sub-Saharan Africa and in Sardinia, while multiple countries of Eastern Europe and the Caucasus have been affected since 2007, chiefly the Russian Federation^5^,^6^,^7^,^8^. The disease was first reported in the Russian Federation in November 2007, after outbreaks in Georgia and Armenia earlier that year, and has remained in the country since then; Azerbaijan briefly reported outbreaks in early 2008, and Armenia reported more cases in 2010 and 2011; Ukraine first reported the disease in July 2012, and has been affected in 2014 and 2015, while cases occurred in Belarus in 2013; four countries have been repeatedly affected since 2014: Lithuania (first report (FR) in January 2014), Poland (FR: February 2014), Latvia (FR: June 2014) and Estonia (FR: September 2014)^9^. In affected countries, the majority of outbreaks in domestic pigs occur in backyard and small-scale farms. The epidemiology of the disease is complex and can involve wild suids (e.g. warthogs, bushpigs, and European boar) and *Ornithodoros spp.* ticks, but African swine fever virus (ASFV) persistence in domestic pig populations is mostly due to the movement of infected pigs or products between farms^5^,^6^,^8^.

In many endemic areas, suspected outbreaks of ASF are usually not reported to authorities. Instead, farmers often sell pigs without apparent signs to traders or at markets in an attempt to limit economic loss (this is also known as “emergency sale”), while pigs with clinical signs are slaughtered on site or sold for slaughter, and dead animals are discarded^5^,^10^,^11^,^12^,^13^,^14^. The decision to sell pigs from affected farms is informed by the clinical diagnosis made by farmers and traders. However, the ability to diagnose ASF in pigs depends on farmers’ awareness of the disease, the clinical signs shown by affected pigs, and the local context (i.e. farmers may be more likely to identify or perceive clinical signs if there are ASF outbreaks in the area). Moreover, due to variation in the infection’s incubation and latent periods, some of the pigs with no sign of disease may also be infected and even infectious.

The objective of this study was to use mathematical modeling to estimate the risk of release of ASF from backyard and small-scale farms via the emergency sale of ASFV infected pigs with undetected clinical signs (also known as “silent release”) and to assess the potential impact of improving the farmers and traders’ clinical diagnosis ability on this risk. Hypothetical scenarios were also used to investigate how the distribution of farms within the small-scale and backyard sectors may influence the silent release of ASFV.

## Results

As ASFV spreads within a pig herd, infected pigs develop clinical signs, which may result in the detection of the disease by the farmer and the sale of animals in which signs of disease have not been noticed. The risk of ASFV release via emergency sale was investigated by simulating an ASF outbreak in a small-scale farm where the farmer’s behavior (i.e. accuracy of disease detection based on clinical diagnosis, and time to detection and emergency sale of animals) may vary.

**Figure 1:**
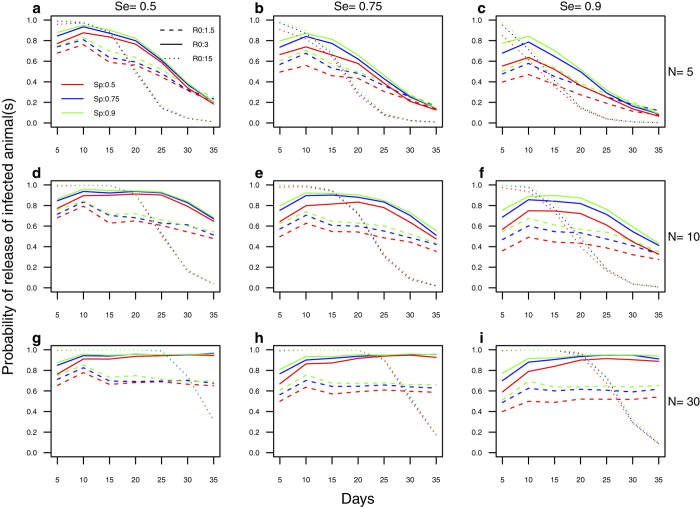
Probability of silent release of ASFV via emergency sale. The three rows show the probability of silent release of ASFV [P(E + L + C) > 0] for N = 5 (**a–c**), N = 10 (**d–f**) and N = 30 (**g–i**). The three panels in a given row show the effect of Se (0.5: a, d, g; 0.75: b, e, h; 0.9: c, f, i) on this probability of silent release. Each panel shows the impact of *R*_0_ (1.5: dashed line; 3: plain line; 15: dotted line) and Sp (0.5:red; 0.75:blue; 0.9:green) on the probability of silent release.

A stochastic individual-based simulation model was used to estimate the probability of releasing ASFV-infected pigs via emergency sale (Fig. 1) for different scenarios of herd size N, disease transmissibility *R*_0_, time T for the farmer to detect the disease and proceed with the emergency sale of animals (also called “time to detection and sale”), and sensitivity (Se) and specificity (Sp) of the farmer or trader’s clinical diagnosis. Se is the probability that the farmer will correctly recognize pigs with clinical ASF, whereas Sp is the probability that pigs without ASF clinical disease will be correctly identified as non-ASF clinical cases. Some pigs may be incorrectly perceived as clinical cases of ASF and sent to the abattoir–for example if they show clinical signs due to another disease, or their behavior was perceived as suggestive of the disease, particularly if the farmer or trader is being influenced by the presence of ASF cases in the same herd or in the local area. The higher *R*_0_, the higher the risk of silent release for short times to detection and sale, but also the faster the risk decreases as the time to detection and sale increases (Fig. 1a–i, dotted lines). For herds of 10 pigs and more, the probability of releasing infected pigs remained high under all scenarios (Fig. 1d–i) except when *R*_0_ = 15 was combined with T ≥ 20 days. For herds of 5 pigs, the risk of silent release decreased for T ≥ 15 days, which reflects that as the disease progresses in a small herd most affected pigs will eventually show clinical signs and be culled or die of the disease (Fig. 1a–c). Improving farmers and traders’ clinical diagnosis ability is not an effective mitigation strategy, as high values of Se and Sp have little effect on the release of ASFV infected animals via emergency sale.

**Figure 2:**
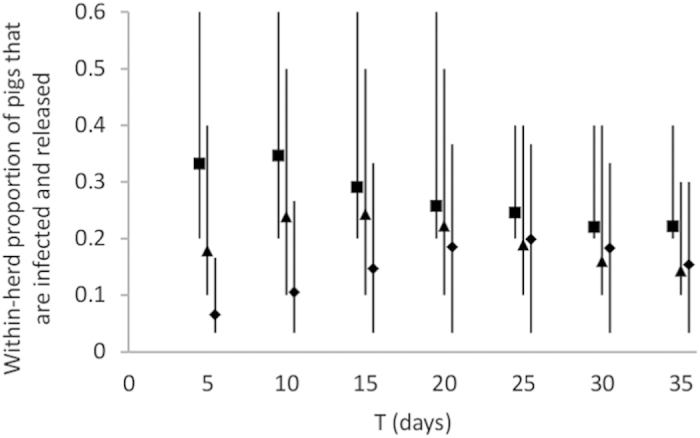
Within-herd proportion of animals that are infected and released via emergency sale. Proportions were computed considering *R*_0_ = 3, Se = 0.9, Sp = 0.9, and for various scenarios of time to detection and sale (T) and herd size N (5: ■, 10: ▲, 30: ♦). Vertical solid lines represent the 95% prediction intervals.

Figure 2 shows the within-herd proportion of pigs that is infected and released via emergency sale (*R*_0_ = 3, Se and Sp = 0.9), for different scenarios of N and T. For herds of 5 pigs, short times to detection and sale are associated with larger proportions of the herd being sold to intermediaries while infected. As the herd size increases, longer times to detection and sale are associated with larger proportions of pigs in ASFV infected herds being released while infected. For example, the largest proportion of infected animals released via emergency sale obtained for herds of 30 pigs was 0.20 for T = 25 days, which corresponds to six ASFV infected pigs being sold to traders or at markets. Delayed reaction of the farmer thus results in larger numbers of infected animals being released in the production sector.

A better understanding of the impact of emergency sale by small-scale and backyard farms could help targeting preparedness and surveillance efforts on the farms that are likely to contribute more to the silent release of ASFV, in particular before official control measures are put in place. To illustrate this point, theoretical scenarios were used to evaluate the average contribution of backyard and small-scale herds of different sizes to the silent release of ASFV infected pigs into hypothetical pig populations during an initial ASF outbreak.

Data on backyard and small-scale farms (defined as farms with 100 pigs or less), obtained from Madagascar^15^ and Senegal^16^, two ASF-endemic countries, as well as thirteen European Union (EU) countries (Eurostat http://epp.eurostat.ec.europa.eu/portal/page/portal/eurostat/home), showed great variation in the distribution of these farms grouped into four size categories (less than 5 pigs, 5–10 pigs, 11–30 pigs, and 31–100 pigs) (see Supplementary Table S1 online). Three profiles were created to represent very distinct distributions of backyard and small-scale farms: Profile 1 has 86% of farms with 1–10 pigs and less than 15% of farms with 31–100 pigs; Profile 2 in contrast has a majority (66%) of farms with 31–100 pigs; and Profile 3 has 70% of farms of intermediate size (i.e. with 5 to 30 pigs).

**Figure 3:**
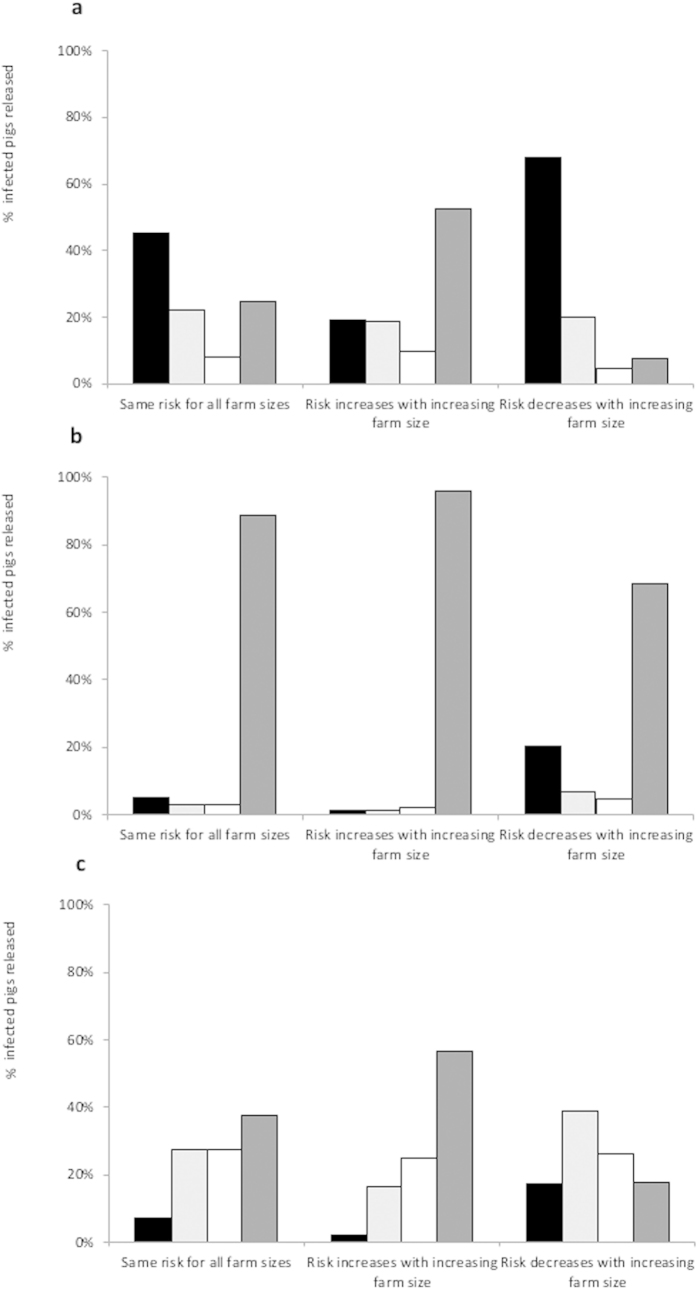
Relative contributions of different categories of herd sizes to the release of ASFV-infected pigs into the production sector via emergency sale. The relative contribution (%) of small-scale and backyard farms of different sizes (N < 5: ■, N = 5–10: 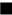, N = 11–30: □, N = 31–100: 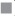) to the release of ASFV-infected pigs was calculated considering a time to detection and sale (T) of 5 days, and for three theoretical profiles of backyard and small-scale production sectors: (**a**) Profile 1 (**b**) Profile 2, and (**c**) Profile 3.

A multi-herd ASF outbreak was assumed to occur in the backyard and small-scale sector, affecting herds as follows: 1) same risk of ASFV infection for all farms, independently of herd size 2) risk of ASFV infection increasing with herd size category, 3) risk of ASFV infection decreasing with herd size category. For each profile, percentages of infected pigs released via emergency sale by each of the four herd size categories of backyard and small-scale herds were then computed. The relative contributions of the four herd size categories to the release of infected pigs, for a time to detection and sale (T) of 5 days was calculated for the three profiles and three relative risk scenarios considered (Fig. 3). Similar results were observed for longer times to detection and sale, except for Profile 1 with a same risk of ASFV infection for all herd sizes, in which case the largest contributors to the silent release of ASFV were farms with less than 5 pigs for T ≤ 10days (36.6–45.2%, Supplementary Figure S1), and farms with 31–100 pigs for T = 15–35days (38.9–46.8%, Supplementary Figure S1). With Profile 1 and a risk of ASFV infection decreasing with herd size, or a similar risk across herd sizes and a short time to detection and sale, farms with less than 5 pigs were the largest contributors to the silent release of ASFV (respectively 67.9% and 45.2% for T = 5d, Fig. 3a). With Profile 2, larger farms (i.e. with 31–100 pigs) contributed most to the release of infected pigs (68.5–95.7%, Fig. 3b), even when the risk of ASFV infection was assumed to decrease with increasing herd sizes. With Profile 3, the relative contributions of the different categories of herds were closer and varied depending on the relative risks of ASFV infection (Fig. 3c), not allowing identifying a group of farms at higher risk of silent release of ASFV. With a risk of ASFV infection increasing with farm size, the majority of infected pigs released via emergency sale were from farms with 31–100 pigs, independently of the small-scale farming profile (52.3%, 95.7% and 56.5% with T = 5d for Profiles 1, 2 and 3, respectively, Fig. 3). The sensitivity analysis performed with a lower sensitivity of the clinical diagnosis (Se = 0.75) for farms with up to 10 pigs showed similar trends in the relative contributions of the four herd size categories to the silent release of ASF (results not shown).

## Discussion

Several authors have suggested that the emergency sale of pigs during ASF outbreaks contributes to the spread of ASF^3^,^6^,^12^,^17^. This finding is supported by the key risk factors for outbreak occurrence identified in endemic areas: movement of pigs^18^, being located in the vicinity of main transportation routes and pig density^17^,^19^. The results presented here are in agreement with these studies, as they show that the emergency sale of pigs by small-scale and backyard farms results in a high probability of release of infected pigs into the production sector. During ASF outbreaks, some farmers with ASF cases in their vicinity may also proceed with the sale of their herd before any disease is detected on their farms, and potentially without their herd being infected with ASFV. The modeling presented here does not apply to such situation, but only to farmers that have detected a clinical case before deciding to sell their herd. When the emergency sale precedes clinical cases in the herd, the risk of ASFV release via emergency sale and the within-herd proportion of infected pigs that are released are expected to be lower than reported here. In addition, this study only investigated the silent release of ASFV via infected pigs sold to traders or on the market. Other practices associated with the emergency sale of affected herds may contribute to disease spread, such as the sale of sick or dead animals^6^,^12^. These mechanisms of transmission were beyond the scope of this analysis because sick or dead animals are not purchased for introduction into swine herds (which is the main mechanism of transmission assumed in this study), but rather, they contribute to disease spread via indirect contacts (e.g. fomites) or contacts between sick and susceptible pigs at markets.

The simulations also suggest that the emergency sale of pigs is a risky practice contributing to ASFV spread regardless of how good farmers are at detecting the disease (in terms of timeliness and/or accuracy). This is because even if clinical detection were perfect, the relatively long incubation period of ASF (median = 5 days, max = 19 days) coupled with the latent period allows for the spread of infected and/or infectious animals before they show clinical signs. Moreover, the unspecific nature of ASF’s initial clinical signs further impedes the accuracy of the clinical diagnosis even for farmers or traders well familiarized with ASF. While shorter times to detection and emergency sale resulted in smaller numbers of infected animals released per infected farm - especially for larger herds - , the probability that infected farms release ASFV-infected pigs is high even for short times to detection and sale. Therefore, enhancing the timeliness of farmers’ action in response to detection is not sufficient to prevent disease spread.

Awareness campaigns for farmers have often been mentioned amongst preventive measures to improve ASF risk management^6^,^20^, together with confinement of pigs, good surveillance systems and laboratory testing in case of suspicion of outbreak. However, although increased awareness as a general principle is desirable, the present study suggests it is unlikely to make an effective contribution to ASF risk management unless adequate financial compensation and support for repopulation following culling provide farmers with strong incentives for reporting ASF suspicions. This is demonstrated by the situation in Russia, where insufficient compensation and consequential lack of cooperation by affected farmers has led to significant underreporting and a large proportion of infected animals being illegally disposed of, slaughtered, or sold^17^,^21^,^22^.

The results from this study need to be interpreted taking into account the following data limitations: at the time the work was conducted, there was very little information on farmer behavior in the pig production systems considered and on the transmissibility of ASFV (i.e., *R*_0_). By using various levels of diagnostic accuracy and a wide range of transmissibility assumptions as part of a sensitivity analysis, this study was able to highlight the risk of silent release of ASFV via emergency sale and the limited influence of enhancing farmers and traders’ clinical diagnosis ability on risk management. Nonetheless, the *R*_0_ values used in this study are in agreement with estimates from recent studies^23^,^24^. Also, the present model focused on the risk of ASFV release only from backyard and small-scale farms, and the findings may thus not be applicable to large farms, where heterogeneous mixing within herds may have to be explicitly modeled^25^.

Backyard and small-scale farms represent the majority of pig farms in most countries^3^,^20^. Given the risk of silent ASFV release from small farms demonstrated here, it is important to further explore their potential role in disease spread so that more effective risk-based prevention, surveillance and control programs can be developed. The FAO recognizes the need to consider the small-scale commercial and backyard sectors in ASF control efforts^22^, and other authors emphasized the usefulness of models to study ASF spread and persistence and assess mitigation strategies^6^. In this context, while the hypothetical scenarios presented here are theoretical and simplified, they illustrate the value of better describing the backyard and small-scale sectors so as to identify the main potential contributors to the silent release of ASFV, thus allowing to better target prevention and surveillance efforts. In Russia, the majority of outbreaks affected the backyard and small-scale commercial farming sectors^8^,^17^,^22^. These farms are considered to be involved in both local and long-distance spread of ASFV^8^,^20^, via illegal movement of pork and pork products, swill feeding, contact between free-range pigs, and emergency sales of pigs. Since 2014, ASF has also been reported in Estonia, Latvia, Lithuania, Poland, and Ukraine^9^. Most cases occurred in the large wild boar populations of the Baltic countries but cases were also reported in pig farms, where the small-scale and backyard sector represents a risk for further ASFV spread. Given that many countries have limited financial resources, coupled with poor cooperation between farmers and veterinary services and therefore significant underreporting, the evaluation of the relative contribution of different farm types to ASFV spread would allow optimizing control efforts. However, this risk distribution is only one component of the overall risk of ASFV spread. Other aspects such as the relative size of the backyard and small-scale sector and the connectivity between farms–both in terms of number and types of links–need to be investigated for countries to be able to develop tailored programs. In addition, socio-economic studies to understand farmers’ disease awareness, behavior, constraints and incentives would also greatly contribute to the development of optimized prevention and control strategies.

In conclusion, enhancing farmers and traders’ clinical diagnosis ability is not sufficient to effectively reduce the high risk of releasing ASFV into the pig production sector via the emergency sale of pigs from affected farms. Unless strong incentives for reporting are provided to farmers, underreporting will occur and emergency sale will contribute to ASFV spread–and persistence within endemic areas. Due to the key role of the small-scale and backyard sectors in ASFV transmission, countries need to identify the farms that are most likely to be infected and facilitate disease spread, in order to better target and optimize prevention and control efforts.

## Methods

### ASFV release from backyard and small-scale farms

**Figure 4:**
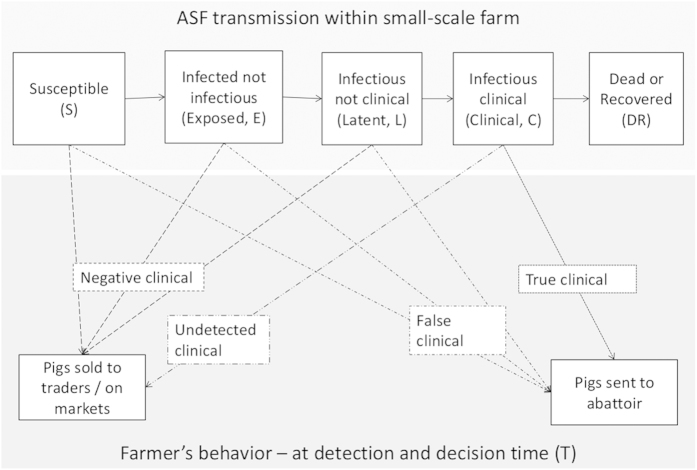
Model structure. The top part shows the successive infectious states of ASFV transmission in a small-scale pig farm. The lower part represents the farmer’s behavior at the time of emergency sale of pigs after the disease has been detected (T). Animals that do not show clinical signs (negative clinical) or whose signs have not been noticed (undetected clinical) are sold to traders or at markets, while the others are sent to the abattoir.

The spread of ASF within a given herd was modeled by dividing the herd into pigs that are susceptible to ASFV infection, and those that are ASFV-infected and in one of the following mutually exclusive states: exposed (infected and not infectious), latent (infectious without apparent clinical signs), clinical (infectious with clinical signs), and recovered or dead (Fig. 4). It was assumed that once the first clinical case appears, it takes a time T (time to detection and sale) for pigs to be sold, either to an abattoir, or to intermediaries (such as traders or markets), depending on the farmer or trader’s clinical diagnosis. Pigs sent to an abattoir are either ASF clinical pigs whose clinical signs have been recognized (“true clinical”), or animals that have been wrongly identified as clinical cases of ASF (“false clinical”), due for example to clinical signs due to other pathogens. Animals correctly identified as non-ASF clinical cases (“negative clinical”), either infected or not, and those that are clinical cases of ASF but which signs have not been noticed by the farmer (“undetected clinical”) are sold to intermediaries via emergency sale. Whether pigs are sold to abattoirs or intermediaries depends on the sensitivity (Se) and specificity (Sp) of the farmer or trader’s clinical diagnosis ability (see details in Supplementary Methods online).

Transmission was assumed to occur from infectious pigs (clinical or latent) to susceptible ones, via contacts made at random (i.e. homogeneous mixing). Infection and progression between disease states occur by chance (see details in Supplementary Methods online), following a Bernoulli infection process and with probabilities defined in Table 1. For example, the time from infection to onset of clinical signs in individual pigs is randomly sampled from the probability distribution of the incubation period of ASF. The probability of any susceptible pig becoming infected was derived from the basic reproduction number *R*_0_ (the average number of secondary cases generated by each infected individual in a totally susceptible population). ASF is a highly infectious disease but at the time of this study the literature on its *R*_0_ was scarce, so the model scenarios assumed *R*_0_ to be in the range 1.5-15, with a most likely value of 3^19^,^26^,^27^,^28^,^29^ (Table 1).

The risk of silent release of ASFV was evaluated for each combination of the following parameters: farm size N with N ∈ {5, 10, 30}, time to detection and sale T with T ∈ {5 days, 10 days, 15 days, 20 days, 25 days, 30 days, 35 days}, and sensitivity Se and specificity Sp of the farmer or trader’s clinical diagnosis ability with (Se, Sp) ∈ {0.5, 0.75, 0.9}. Scenarios of Se and Sp represented low to high accuracy of the farmer or trader’s clinical diagnosis ability, in order to test the following hypotheses: 1) a higher Se would result in more clinical cases of ASF being sold to abattoir and thus a lower risk of release of ASFV; 2) the lower the Sp, the more non-clinical cases of ASF would be sold to abattoirs rather than to traders or at markets, resulting in a lower risk of ASFV release.

Each simulation was run until ASF was detected by the farmer and emergency sale occurred (i.e. throughout the time to detection and sale, T). The status of each pig in the herd was simulated daily over this simulation period, during which pig herds were assumed to have a closed population.

Implementation of the model and analyses of results were conducted using the R statistical programming language environment version 2.14.1 (http://cran.r-project.org/). For each scenario and parameter combination, 1500 iterations were run. The risk of silent release of ASFV via emergency sale was measured by the probability of selling at least one infected pig to intermediaries, as well as by the proportion of the herd that is infected and sold to intermediaries (see details in Supplementary Methods online).

### Contribution of farms of different sizes to the release of infected animals into the production sector

For the hypothetical scenarios evaluating the relative contribution of various sizes of backyard and small-scale herds to the silent release of infected pigs, we considered an initial ASF outbreak affecting multiple herds in the backyard and small-scale sector. The simplified scenarios did not attempt investigating the role of emergency sale in ASFV spread beyond this initial outbreak. Moreover, no statement was made as to the size of the outbreak (i.e. the number of affected farms), and the analysis only focused on the average contribution of the four herd size categories to the silent release of ASFV. However, with smaller outbreaks, more variation in these relative contributions should be expected than with large outbreaks.

The distribution of affected herds across the four herd size categories was determined based on the relative percentages of herds in each herd size category (defined by three “Profiles”) and the relative risks of ASFV infection of each category. The three profiles of backyard and small-scale sector are presented in Table 2, while the different scenarios of relative risk of infection are summarized in Table 3. To determine the mean percentages of infected pigs released via emergency sale by each of the four herd size categories, the relative distribution of infected herds across herd sizes was combined with the mean within-herd proportion of infected pigs released via emergency sale. These within-herd proportions of infected pigs released were assumed similar to the model estimates with Se and Sp = 0.9, but a sensitivity analysis was conducted with a lower sensitivity (Se = 0.75) for farms with up to 10 pigs.

For all hypothetical scenarios described above, it was also assumed that: all farmers with an infected herd proceeded with the emergency sale of animals, that their decision to sell pigs was based on clinical diagnosis, that the within-herd proportion of infected pigs released via emergency sale was the same for all herds in a size category, and that farmers neither implemented control measure nor reported a suspected outbreak during the initial ASF outbreak.

## Additional Information

**How to cite this article**: Costard, S. *et al.* Small-scale pig farmers′ behavior, silent release of African swine fever virus and consequences for disease spread. *Sci. Rep.* **5**, 17074; doi: 10.1038/srep17074 (2015).

## Supplementary Material

Supplementary Information

## Figures and Tables

**Table 1:** Model parameters and probability distributions used to simulate within-herd ASFV spread.

Parameter and symbol	Value	Source
Basic reproduction number (*R*0)	1.5, 3, 15	Assumption^19^,^26^,^27^,^28^,^29^
Incubation period (from exposed to clinical, *T*_*EC*_), days	2+ Weibull (1.092, 4.197 (Median: 5, Range: 2–19)	30,31,32,33
Latent period (from latent infectious to clinical, *T*_*LC*_), days	Uniform(1,2)	30,32
Time from infection to onset of infectiousness, days	*T*_*EC*_*– T*_*LC*_	
Duration of disease (*D*), days	5+ Weibull(1.104, 6.271) (Median: 9.5, Range: 5–30)	30,31,32,33

**Table 2:** Distribution of small scale and backyard farms in the three hypothetical sector profiles used to illustrate the relative contributions of different categories of herd sizes to the release of ASFV-infected pigs during an initial ASF outbreak.

	Distribution (%)		
Herd size	Profile 1	Profile 2	Profile 3
<5 pigs	66%	20%	15%
5–10 pigs	20%	7%	35%
11–30 pigs	7%	7%	35%
31–100 pigs	7%	66%	15%

**Table 3:** Hypothetical scenarios of relative risks of ASFV infection used to illustrate the relative contributions of different categories of herd sizes to the release of ASFV-infected pigs during an initial ASF outbreak.

	Relative risk of ASFV infection		
Herd sizecategories	Same risk forall farm sizes	Risk increases withincreasing farm size	Risk decreases withincreasing farm size
<5 pigs	1	1	5
5–10 pigs	1	2	3
11–30 pigs	1	3	2
31–100 pigs	1	5	1
