# Supplementary material for: Small-scale pig farmers’ behavior, silent release of African swine fever virus and consequences for disease spread
Source: Sci Rep. 2015 Nov 27;5:17074. doi: 10.1038/srep17074 (PMC4661460; doi:10.1038/srep17074)
Supplement: Supplementary Information [file srep17074-s1.pdf]

Small-scale pig farmers' behavior, silent release of African swine fever virus and consequences for disease spread

Solenne Costard, Francisco J. Zagmutt, Thibaud Porphyre, Dirk Udo Pfeiffer

Supplementary Methods

Here we provide further information about the model structure and the calculation of its outputs.

Model description

ASFV transmission. A stochastic, individual-based, discrete-time state-transition model was developed to simulate the spread of ASFV through a small-scale pig herd (main document, Fig. 4). All pigs are initially susceptible (S) and are assumed to have equal risk of infection once a first pig is infected with ASFV. An infected pig is first infected but not yet infectious (Exposed, E), and subsequently becomes infectious but not showing clinical signs (Latent, L) and then infectious with clinical signs (C) until it either recovers or dies (DR). The time step used in the model was 1 day.

All animals were considered equally susceptible and there was no immunity to ASFV at the time an infected animal was introduced into the herd. Also, all animals were fully infectious during the infectious period (both latent and clinical states) and developed clinical signs. After developing the disease, pigs could either die or recover and become resistant to re-infection by ASFV during the whole duration of the outbreak. Pigs of a same herd were assumed to mix randomly, and the same transition risks were assumed to apply to all animals in the herd.

The continuous-time analogue of our model of transmission dynamics of ASF within a given pig herd can therefore be expressed by the following equations:

$$\frac{dS}{dt} = -\beta(E+C)S$$
$$\frac{dE}{dt} = \beta(E+C)S - f_1 E$$
$$\frac{dL}{dt} = f_1 E - f_2 L$$
$$\frac{dC}{dt} = f_2 L - dC$$
$$\frac{dDR}{dt} = dC$$

where β is the transmission coefficient, f_1, is the risk of an animal becoming infectious without signs between time *t* and *t+1*, f_2 is the risk of an infected animal becoming infectious

with signs between time *t* and *t+1* and *d* is the risk of an animal dying or recovering between time *t* and *t+1*.

Because the model operates in discrete time and involves populations of various sizes, the probability that a susceptible pig becomes infected at a given time step was approximated by a Binomial distribution, such as:

$$P\inf_{t+1} = 1-(1-p)^{(L_t+C_t)}$$

In this study, we assumed transmission being density-dependent, so that *p*, the probability of an effective contact between any two pigs in each time step, is given by:

$$p = \frac{k}{N-1}$$

where *N* denotes the herd size. This is to reflect the fact that when the herd size increases, the farm density also increases and so does the probability of contacts between pigs [1].

The effective contact rate, *k*, was defined as

$$k = \frac{R_0}{D}$$

where *D* is the duration of the disease and R_0 is the basic reproduction number. R_0 corresponds to the number of secondary infections caused by a single infected individual introduced into a totally susceptible population [2].

Herd sizes (N) of 5 and 10 pigs were used to represent backyard and traditional small-scale farms, and 30 pigs were used for small-scale semi-commercial farms.

Due to the paucity of information on the transmissibility of ASF, 3 scenarios of R_0 were considered in this study, selected on the basis of estimates for other highly contagious animal diseases. Firstly, the minimum and maximum plausible values for *R0* were set using estimates for classical swine fever (CSF), because of its epidemiological similarities with ASF for pig-to-pig transmission. The values of 1.5 and 15 were derived from two studies [3,4]. Secondly, the value of 3 was selected as the most likely R_0 for ASF. This is because a number of studies for CSF, avian influenza or foot-and-mouth disease, other highly transmissible diseases, yielded R_0 around 3 [3,5-9]

For each infected animal, the transition times from each infectious stage to the next depends on the incubation period, latent period, and duration of disease for ASFV. As pigs are considered to become infectious 1 to 2 days before they become clinically diseased, a Uniform(1,2) distribution was used to represent the time from latency to onset of clinical signs. For both incubation period and duration of disease, Weibull distributions were used because they allow to model time until occurrence of an event when the probability of

occurrence changes with time [10] and its distribution is right-skewed. Both incubation period and disease duration were formulated using a constant element (to set the minimum duration of state) and a stochastic one (Weibull(shape, scale)) for determining the duration of the state according to values reported in the literature (see main text, Table 1).

Farmer's behavior and emergency sale of pigs. Factors related to the farmer's reaction in case of ASF outbreak were included in the model. Firstly, the time to detection and sale, T, represents the time taken by farmers to detect clinical signs in affected pigs and cull or sell animals. It is modeled as the time between the day a first animal starts showing clinical signs and the day the farmer proceeds with the emergency sale of his swine herd. Times T of 5 to 35 days represented potential variation in the time taken by farmers to detect clinical signs in affected pigs and proceed with the emergency sale of pigs [11,12].

Secondly, the ability of farmers and/or traders to correctly identify animals with clinical ASF was represented by the sensitivity and specificity of his clinical diagnosis. The sensitivity of the farmer or trader's clinical diagnosis, Se, is the probability that farmers/traders will notice clinical signs in pigs that have declared the disease (i.e. pigs in the infectious clinical stage, C) and send them to slaughter. The specificity of the clinical diagnosis, Sp, is the probability that pigs without ASF clinical disease will be correctly identified as non-ASF clinical cases,. Some pigs may be wrongly identified as clinical cases of ASF (false clinical), either because they show clinical signs due to other diseases or because their (non-sickly) behavior was perceived as a sign of the disease – the judgment of the farmer or trader being influence by the local context and presence of at least one clinical case on the farm. Animals correctly recognized as non-ASF clinical cases (negative clinical), either susceptible or infected, and clinical pigs in which signs have not been noticed by the farmer or trader (undetected clinical) are sold to traders or on markets (emergency sale) (main document, Fig. 4). Among these animals, the infected and infectious ones will contribute to the further spread of the disease. Se and Sp values of 0.5, 0.75 and 0.9 were used to represent low to high accuracy of the farmer or trader's clinical diagnosis ability. With Se and Sp = 0.5, animals have a 50% chance of being correctly diagnosed as clinical or non-clinical, while for values of 0.9 they have a 90% chance of being correctly diagnosed. Due to the non-specific nature of ASF clinical signs, especially in the early stages of the disease, values closer to 1 were not used.

At time T, each animal of the herd has a Binomial probability of being sent to the abattoir. In the case of clinically infected animals (C), the success probability of the Binomial distribution is equal to Se. For non-clinical animals (S, E and L), this success probability is equal to: 1 – Sp. Animals that are not sent to the abattoir are sold to traders or at markets. Each simulation stops right after the emergency sale is performed. As described in the main text, different scenarios were implemented to assess the effect of the three factors on the effectiveness of the control strategy.

Model outputs

Due to the stochastic nature of the model, simulation was replicated for each scenario and parameters combination until a stable average behavior was reached (n = 1500). All analyses and implementation of the model were conducted in R version 2.14.1 [13].

The risk of release of ASFV from backyard and small-scale farms via the emergency sale of pigs was characterized by the probability that at least one infected animal remained at the end of the simulation and was sold to intermediaries (P(E+L+C)>0). This probability was also called the probability of release of at least one infected animal, or the probability of silent release, and was calculated across the 1,500 simulations run for each scenario.

The proportion of animals in a herd that was infected and released, $p_{\inf}$, was calculated at the end of each simulation for the different scenarios considered:

$$p_{\inf} = \frac{E + L + I}{N}$$

References

1 White, R. G. & Vynnycky, E. *An Introduction to Infectious Disease Modelling*. 1st edn (Oxford University Press, USA, 2010).

2 Anderson, R. M. & May, R. M. *Infectious Diseases of Humans: Dynamics and Control*. 768 (Oxford University Press, USA, 1992).

3 Stegeman, A., Elbers, A. R., Bouma, A., de Smit, H. & de Jong, M. C. Transmission of classical swine fever virus within herds during the 1997-1998 epidemic in The Netherlands. *Prev. Vet. Med.* **42**, 201-218, (1999).

4 Howard, S. C. & Donnelly, C. A. Estimation of a time-varying force of infection and basic reproduction number with application to an outbreak of classical swine fever. *J Epidemiol Biostat* **5**, 161-168, (2000).

5 Bos, M. E. et al. Back-calculation method shows that within-flock transmission of highly pathogenic avian influenza (H7N7) virus in the Netherlands is not influenced by housing risk factors. *Prev. Vet. Med.* **88**, 278-285, (2009).

6 Eble, P., de Koeijer, A., Bouma, A., Stegeman, A. & Dekker, A. Quantification of within- and between-pen transmission of Foot-and-Mouth disease virus in pigs. *Vet. Res.* **37**, 647-654, (2006).

7 Ward, M. P., Maftei, D., Apostu, C. & Suru, A. Estimation of the basic reproductive number (R_0) for epidemic, highly pathogenic avian influenza subtype H5N1 spread. *Epidemiol. Infect.* **137**, 219-226, (2009).

8 Haydon, D. T., Woolhouse, M. E. & Kitching, R. P. An analysis of foot-and-mouth-disease epidemics in the UK. *IMA J. Math. Appl. Med. Biol.* **14**, 1-9, (1997).

9 Tiensin, T. et al. Transmission of the highly pathogenic avian influenza virus H5N1 within flocks during the 2004 epidemic in Thailand. *J. Infect. Dis.* **196**, 1679-1684, (2007).

10 Vose, D. *Risk Analysis: A Quantitative Guide*. 3rd edn (John Wiley & Sons, Ltd, 2008).
11 Food and Agriculture Organization of the United Nations. African swine fever in the Russian Federation: risk factors for Europe and beyond. *EMPRES Watch* **28**, 1-14, (2013).
12 Nigsch, A., Costard, S., Jones, B. A., Pfeiffer, D. U. & Wieland, B. Stochastic spatio-temporal modelling of African swine fever spread in the European Union during the high risk period. *Prev. Vet. Med.* **108**, 262-275, (2013).
13 R: A language and environment for statistical computing (R Foundation for Statistical Computing, Vienna, Austria, 2011).

Supplementary Tables

Supplementary Table S1: Distribution of small scale and backyard farms (herdsize ≤100) in selected countries of the European Union, Madagascar and Senegal.

Country	Percentage of farms with ≤100 pigs	Herd size distribution (%)			
		< 5	5-10	11-30	31-100
Bulgaria	100	95	4	1	1
Denmark	16	20	12	22	46
France	64	68	17	6	9
Germany	61	29	21	18	32
Hungary	100	76	18	4	3
Italy	97	75	16	4	4
Latvia	99	75	19	3	3
Lithuania	100	75	23	1	1
Netherlands	10	20	10	9	62
Poland	87	44	29	15	13
Romania	100	89	10	1	0
Spain	86	66	23	5	6
United Kingdom	72	45	24	15	17
Madagascar	100	60	20	17	3
Senegal	99	12	30	42	15

Supplementary Figures

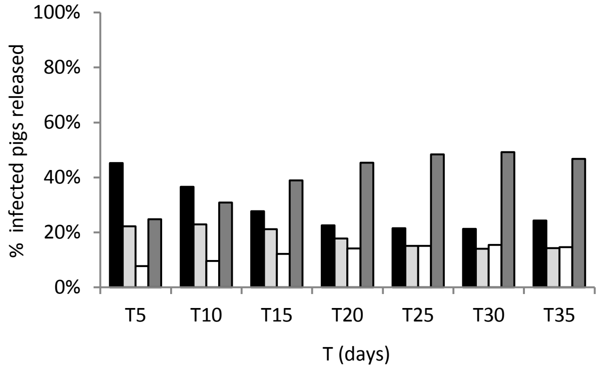

Supplementary Figure S1: Relative contribution of different categories of herd sizes to the risk of the ASF release for Profile 1. The relative contribution (%) of small-scale and backyard farms of different sizes (N<5: ■, N=5-10: ■, N=11-30: □, N=31-100: ■) to the release of ASFV-infected pigs was calculated for Profile 1, for different times to detection and sale (T).
